# The intracellular signalosome of PD-L1 in cancer cells

**DOI:** 10.1038/s41392-018-0022-9

**Published:** 2018-09-28

**Authors:** David Escors, María Gato-Cañas, Miren Zuazo, Hugo Arasanz, María Jesus García-Granda, Ruth Vera, Grazyna Kochan

**Affiliations:** 1 Navarrabiomed, Complejo Hospitalario de Navarra, IdISNA, Irunlarrea 3, 31008 Pamplona, Navarra Spain; 2 Rayne Institute, Division of Infection and Immunity, University College London, 5 University Street, WC1E 6JF London, UK; 3 Oncology Department, Complejo Hospitalario de Navarra, IdISNA, Irunlarrea 3, 31008 Pamplona, Navarra Spain

## Abstract

Programmed cell death-1 ligand-1 (PD-L1) overexpression in cancer cells accelerates tumor progression. PD-L1 possesses two main pro-oncogenic functions. First, PD-L1 is a strong immunosuppressive molecule that inactivates tumor-specific T cells by binding to the inhibitory receptor PD-1. Second, PD-L1 function relies on the delivery of intrinsic intracellular signals that enhance cancer cell survival, regulate stress responses and confer resistance toward pro-apoptotic stimuli, such as interferons. Here, we review the current knowledge on intracellular signal transduction pathways regulated by PD-L1, describe its associated signalosome and discuss potential combinations of targeted therapies against the signalosome with PD-L1/PD-1 blockade therapies.

## Cancer therapies: More to PD-L1 than meets the T cell

Programmed cell death-1 ligand-1 (PD-L1) favors tumor progression by suppressing the immune system and regulating pro-survival signaling pathways in cancer cells. Therapeutic antibodies that block the interaction between PD-L1 expressed in cancer cells and the PD-1 receptor on T cells are showing great promise for treating many human cancers. However, remarkably little is known about the effects of PD-L1 in cancer cells themselves. Grazyna Kochan at the Instituto de Investigación Sanitaria de Navarra, Spain, and colleagues review current knowledge on PD-L1 expression and function in cancer cells. PD-L1 can enhance cancer cell survival by regulating glucose metabolism and conferring resistance to pro-apoptotic stimuli, such as interferons, without engaging PD-1. Further understanding of the intracellular signaling pathways that are regulated by PD-L1 could inform on therapeutic combinations that could enhance the efficacy of anti-PD-L1/anti-PD-1 antibodies.

## Introduction

The concept of using immunotherapies to fight cancer was supported until recently by a few immunologists and oncologists who were convinced of their potential to eliminate cancer and their metastases. However, most oncologists were convinced that cancer could only be effectively treated with radiotherapy, classical chemotherapy, and kinase inhibitors (targeted therapies). In fact, slightly more than a decade ago, oncologists and pharmaceutical companies devoted major efforts and resources to the development of novel small molecules and little time to immunotherapies.

In 2012, a major turning point occurred following the publication of encouraging results from clinical trials conducted by Dr. Suzanne Topalian using antibodies that blocked the immunosuppressive programmed death 1 ligand 1 (PD-L1)/programmed death 1 (PD-1) interactions.^1,2^ Indeed, these trials showed therapeutic efficacies without precedent over a wide range of cancers with possibly the exception of ipilimumab (a CTLA4-specific antibody), developed by Professor James Allison’s team.^3^

Systemic administration of PD-L1/PD-1 blocking antibodies results in a strong potentiation of the anti-tumor capacities of T cells, as many preclinical studies have shown for some time.^4–7^ Since 2012, PD-L1/PD-1 blockade therapies have proven efficacious for the treatment of many human cancers. Pembrolizumab was the first PD-L1/PD-1 blocking agent to be approved by the FDA, being granted the designation of breakthrough therapy for malignant melanoma in 2014.^8^ Other PD-L1/PD-1 blocking antibodies, including nivolumab, atezolizumab, durvalumab and avelumab, have been approved for clinical use.^9–13^ In 2017, pembrolizumab was the first FDA-approved immunotherapeutic agent for the treatment of solid tumors with unresectable mismatch-repair deficiency and microsatellite instability.^14^

Thus, presuming that substantial amounts are known about the mechanisms of action of PD-L1/PD-1 interactions and how T cell and cancer cell responses are regulated by these interactions is logical. However, this is far from reality. The clinical use of PD-L1/PD-1 blockade agents is advancing far past basic mechanistic studies. Although this might be practical from the point of view of the patient, the lack of knowledge on how these interactions work can lead to several missed opportunities for therapeutic interventions. Here, we review the current knowledge on PD-L1 signal transduction pathways, describe the intracellular signalosome of PD-L1 in human cells and discuss the potential use of targeted therapies that would inhibit PD-L1-dependent pathways in cancer cells.

## PD-L1/PD-1 regulation and anti-tumor immunity

**Fig. 1:**
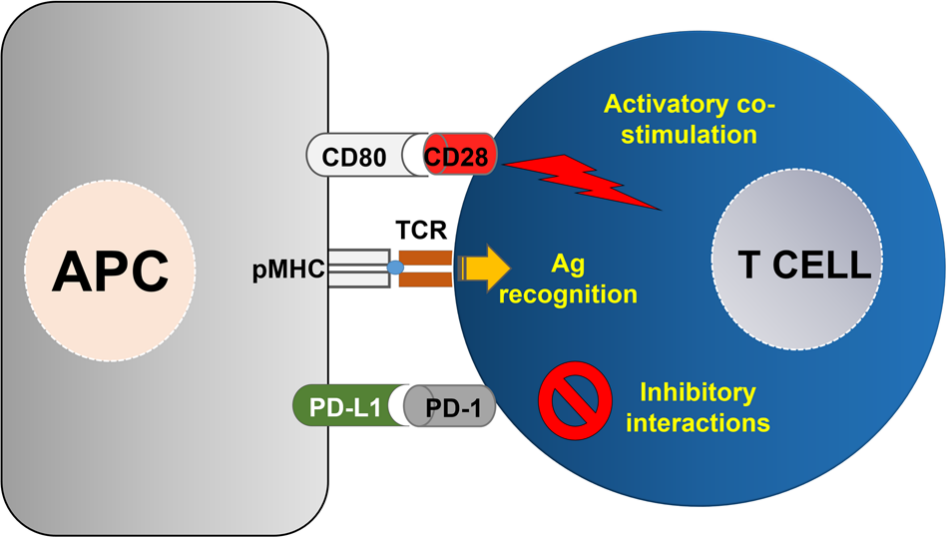
T cell activation relies on antigen recognition and co-stimulatory/inhibitory interactions. On the left, an antigen-presenting cell (APC) is represented, presenting antigen complexed to MHC molecules (pMHC) to a T cell shown on the right. The T cell binds to the pMHC via the T cell receptor (TCR) and establishes stimulatory, as well as inhibitory interactions, represented by CD80-CD28 binding (top) and PD-L1/PD-1 (bottom), respectively. The integration of all these intracellular signals determines the level of T cell activation

Without doubt, T lymphocytes are the main effector anti-tumor cells of acquired immunity. T cells recognize potentially antigenic peptides from pathogens presented to them by antigen-presenting cells (APCs). Some of these are professional APCs that include mostly cells of the myeloid lineage, such as dendritic cells (DCs) and macrophages, which capture and process antigens into antigenic peptides. These peptides are bound to major histocompatibility complex molecules (MHCs) that are exposed to the cell surface to be recognized by T cell receptors (TCRs). In addition to TCR-peptide-MHC binding, T cells require further interactions known as “co-stimulation” to achieve the correct activation state and proliferate (Fig. 1). Many of these interactions are delivered to the T cell by the B7 family of molecules expressed on APCs,^15^ classically represented by CD80 (B7-1) and CD86 (B7-2). These bind to CD28 on T cells and provide activating co-stimulation to the T cell during antigen recognition at the immunological synapse (Fig. 1). These signals rescue T cells from apoptosis and stimulate the proliferative signals transmitted by the TCR.

In 1999, an additional member of the B7 family was discovered, named B7 homolog 1 (B7-H1), that engaged T cells during antigen presentation but caused IL-10 secretion instead of Il-2 production.^16^ This result strongly suggested that in contrast to CD80 or CD86, B7-H1 plays a role in suppressing T cell responses. In 2000, its receptor on T cells was identified to be PD-1, and B7-H1 was also known as PD-L1^17^ (Fig. 1). Since then, the immunosuppressive properties of PD-L1/PD-1 interactions and their physiological role in keeping systemic immunotolerance toward autoantigens have been extensively demonstrated.^18^ PD-L1 is expressed constitutively in myeloid cells and inducibly in many cell types after exposure to pro-inflammatory stimuli. Furthermore, dysregulated PD-L1/PD-1 interactions were demonstrated to contribute to several pathologies, for example, by maintaining T cell exhaustion in chronic viral infections and their participation in the onset of autoimmune diseases.^19,20^ Importantly, many tumors in vivo and cancer cell lines overexpress PD-L1, contributing to the strong inhibition of anti-cancer T cell responses in preclinical models and human neoplastic disease.^21^ Therefore, PD-L1 overexpression in tumors was generally found to be an indicator of progression and poor prognosis in cancer.

Most studies have addressed PD-L1/PD-1 interactions over T cell functions and TCR signal transduction, but only a few have concentrated on the intrinsic signaling of PD-L1 molecules in PD-L1-expressing cells. Recent published evidence from a few research groups, including ours, has suggested that PD-L1 delivers intrinsic pro-survival signals to cancer cells that favor tumor progression.

## Molecular structure of PD-L1 and regulation of its expression

**Fig. 2:**
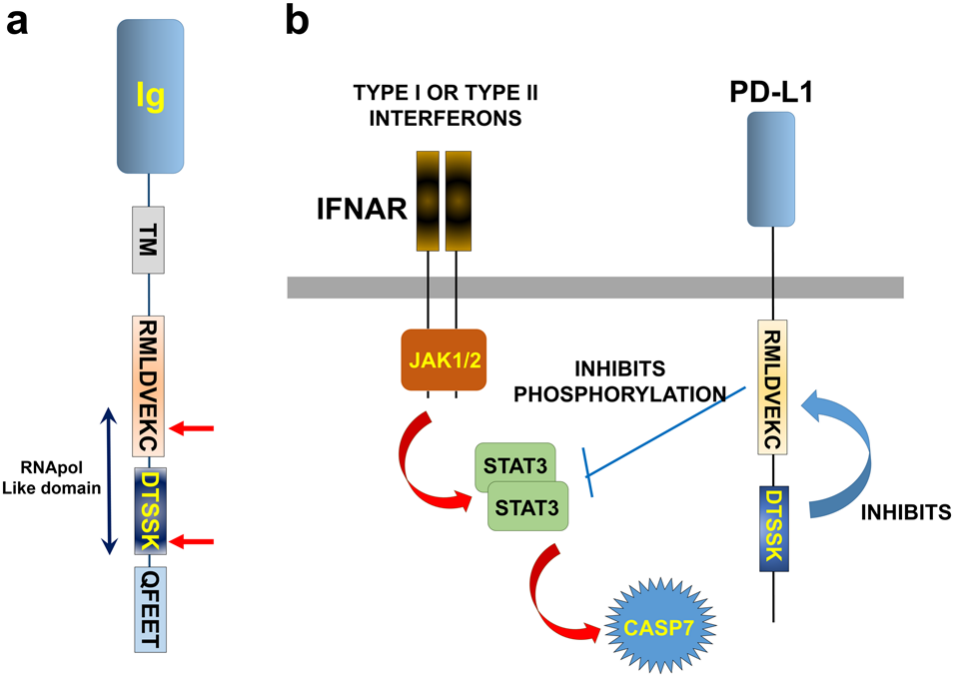
PD-L1 domains and crosstalk with interferon signaling in cancer cells. **a** The domain structure of PD-L1 is represented. Ig extracellular immunoglobulin domain, TM transmembrane domain. The RMLDVEKC, DTSSK, and QFEET motifs are represented in the intracytoplasmic region of PD-L1. The RNA pol-like motif identified by MotifFinder, containing part of the RMLDVEKC motif and the entire DTSSK motif is indicated. Red arrows indicate the inhibitory lysines in the murine PD-L1 molecule. **b** The mechanism by which PD-L1 counteracts interferon-mediated apoptosis is represented. A function associated with the RMLDVEKC motif is required to inhibit STAT3 phosphorylation, which, in turn, halts caspase-mediated apoptosis. The DTSSK motif acts as a negative regulator of the RMLDVEKC motif

The molecular organization of PD-L1 is similar to that of other B7 molecules and typical of the immunoglobulin superfamily. PD-L1 is a type I transmembrane glycoprotein that adopts an immunoglobulin structure with an Ig variable (V) distal region and an Ig constant (C) proximal region in its extracellular domain (Fig. 2a). The V sequence presents a standard Ig-like domain with complementary determining-like regions (CDRs) that form the binding domain to PD-1 in a 1:1 stoichiometry, similar to antigen recognition by antibodies and TCRs.^22,23^ PD-L1 is anchored to the cell membrane by a hydrophobic transmembrane sequence, followed by a short intracytoplasmic region with very poor sequence similarity to that of other B7 molecules. Nevertheless, this intracellular region contains three sequences that are conserved in mammalian PD-L1 molecules, the RMLDVEKC, DTSSK and QFEET motifs (Fig. 2a). There is accumulating evidence that this intracytoplasmic region transduces survival signals, most likely mediated by functions associated with the RMLDVEKC and DTSSK motifs, as we recently showed.^24,25^ The intracytoplasmic domain of the murine PD-L1 contains two lysine residues that may become ubiquitinated and therefore regulate PD-L1 stability and signal transduction.^25,26^

PD-L1 is constitutively expressed at varying levels in cells of the myeloid lineage, such as DCs, macrophages, and myeloid-derived suppressor cells (MDSCs) but also in other cell types.^5,18,27–33^ This includes many tumors and cancer cell lines.^7,21^ Moreover, PD-L1 is up-regulated in many cell types, including cancer cells, by a range of pro-inflammatory stimuli.^26,34–36^ This expression is regulated via the binding of transcription factors to its promoter activated by pro-inflammatory cytokines. For example, interferon gamma (IFNγ) produced by T cells activates the Janus kinase (JAK) signal transducer and activator of transcription (STAT) pathway, resulting in transcriptional activation of interferon regulatory factor 1 (IRF1), which then binds to the PD-L1 promoter.^37^ Tumor necrosis factor alpha (TNFα) and IFNγ also activate the NF-κB pathway that can also transcriptionally transactivate PD-L1 transcription. All these pathways provide a mechanistic explanation for the high expression levels of PD-L1 associated with inflamed tissues, including highly infiltrated tumors (“hot” tumors).^38^ Nevertheless, the regulation of PD-L1 transcription also differs depending on the cell type and physiological and pathological situation. PD-L1 expression is regulated by Sox2 in hepatocellular carcinoma,^39^ STAT3 in human glioma^40^ and STAT1 in multiple myeloma.^36^ The various mechanisms that control PD-L1 expression reflect its differing roles depending on the location and cell type.

Many cancer cells show constitutively high levels of PD-L1 expression, which is explained by the oncogenic activation of classical pathways, such as the rat sarcoma (Ras), AKT-molecular target of rapamycin (mTOR), epidermal growth factor receptor (EGFR), mitogen-activated protein kinase kinase (MEK)-extracellular signal regulated kinase (ERK) and mitogen-activated protein kinase (MAPK) p38.^41–45^ Other pro-oncogenic pathways have been recently linked to PD-L1 expression in several cancer types.^46,47^ PD-L1 up-regulation in cancer cells by the dysregulated activation of pro-oncogenic pathways possibly helps cells adapt to strong pro-inflammatory environments by counteracting the immune system. In addition, hypoxia and epigenetic mechanisms also present within the tumor environment regulate PD-L1 expression.^48–50^ For example, the expression of microRNA 513 down-modulates PD-L1 mRNA translation in human cholangiocytes,^51^ while microRNA 152 performs a similar function in gastric carcinoma.^52^ Several cancer cell types disrupt the structure of the PD-L1 mRNA 3′UTR to constitutively increase PD-L1 expression.^53^

It was recently demonstrated that PD-L1 stability and functions can be regulated by interactions with other membrane proteins, such as CMTM6 and CMTM4. Interestingly, these two type III transmembrane proteins with previously unknown functions specifically associate with PD-L1 and inhibit its ubiquitination. Thus, PD-L1 stabilization and increased surface levels potentiate the T cell inhibitory functions of PD-L1, favoring immune escape.^54,55^

## PD-L1 and tumor progression

It was assumed for a long time that the failure of immunotherapies was caused by the intrinsic poor immunogenicity of cancer cells. However, a few years ago, tumors were demonstrated to be quite immunogenic.^56^ Indeed, arising neoplastic lesions are usually immunogenic, but poorly immunogenic cancer cell variants are strongly selected by the immune system by eliminating the most immunogenic cell clones. These selected variants are those that finally progress and comprise the tumors at the time of diagnosis. This selection process was termed cancer immunoediting.^57–60^ Interferons produced by effector immune cells that infiltrate tumors constitute a major driving force of immunoediting. Interferons exhibit strong cytotoxic and anti-proliferative activities that eliminate the most immunogenic and less-resistant cancer cells, leaving the most resistant variants to proliferate.^58–60^

One way for cancer cells to escape from the immune attack is to directly inhibit effector cytotoxic cells using PD-L1/PD-1 interactions. In addition, PD-L1 can directly deliver intracellular anti-apoptotic signals to cancer cells, helping them survive IFN cytotoxicity.^25^ PD-L1 is frequently overexpressed in many tumors, aided by its transcriptional up-regulation by IFNs. Therefore, high tumor PD-L1 expression has been shown to be a marker of a poor prognosis for many but not all cancer types.^61–67^ PD-L1 can be expressed in cancer cells, stromal cells and immune cells, including infiltrating myeloid and T cells. Some studies have indicated that PD-L1 expression in tumor cells is sufficient for tumor progression,^68^ while others have claimed that PD-L1 expression in other tumor-associated cell types is important.^69–71^ Therefore, although there is a general consensus on the association of PD-L1 expression with tumor progression,^72–75^ many other factors influence the therapeutic outcome of classical therapies and immunotherapies.^76^ The role of PD-L1 in tumor progression is a very active subject of research that is outside the scope of this commentary and has been extensively reviewed elsewhere.^77^

The clinical application of PD-L1/PD-1 blockade therapies was thought to prevent tumor progression by “removing the breaks” in T cells.^1^ However, other factors apart from “removing breaks” in T cells influence the efficacy of PD-L1/PD-1 blockades, including interferon signatures within the tumor.^78–80^ Indeed, a functional interferon signal transduction pathway in cancer cells is required for the clinical efficacy of PD-L1/PD-1 blockade agents. Patients with tumors in which the interferon signal transduction pathway has been inactivated by somatic mutations are refractory to PD-1 blockade therapies.^81,82^ In fact, apart from PD-L1 up-regulation, hyperactivated PD-L1 mutants with enhanced signal transduction capacities are selected by cancer cells to interfere with the pro-apoptotic branches of interferon signal transduction pathways.^25^

## PD-L1 signal transduction pathways in cancer cells

Most studies on the participation of PD-L1 in tumor progression are based on its T cell inhibitory activities via binding to PD-1. Thus, PD-1 engagement with PD-L1 interferes with TCR signal transduction and co-stimulation by recruiting the SHP-1 and SHP-2 phosphatases to the intracellular domain of PD-1^83,84^ and up-regulating the expression of CBL E3 ubiquitin ligases to induce TCR down-modulation.^5,85,86^ However, the direct protective role of PD-L1 expression in cancer cells has thus far been neglected, thereby missing an opportunity for targeted therapies that directly interfere with PD-L1 signal transduction pathways in cancer cells.

In 2004, PD-L1-expressing cancer cells were found to be significantly more resistant to T cell cytotoxicity by providing a protective molecular shield that inhibited their activities.^87^ It was also shown that PD-L1/PD-1-blocking antibodies could break this shield in cell cultures, restoring T cell cytotoxicity.^4^ The first evidence of the intrinsic signaling activities of PD-L1 independently of its function as a PD-1 engager was published in 2008. An alternative explanation for the “PD-L1 molecular shield” was provided in which PD-L1 directly conferred cancer cell resistance against pro-apoptotic stimuli.^24^ It was then proposed that PD-L1 transmitted protective signals to cancer cells. The authors showed that P815 and Renca cell cancer cell lines required PD-L1 expression to resist the T cell attack. This resistance was abrogated with anti-PD-L1 antibodies, as expected. However, cancer cells remained resistant to cytotoxicity even if T cells expressed a signal-null PD-1. These results clearly indicated that PD-L1 in cancer cells directly conferred resistance to T cell-mediated death without relying on the PD-1-dependent inhibition of T cells. This was further proven by demonstrating that a PD-L1 molecule without a functional intracytoplasmic domain lost its protective capacities. Furthermore, PD-L1 expression also interfered with a range of pro-apoptotic signals, such as first apoptosis signal receptor (Fas)-Fas ligand (FasL) interactions or pro-apoptotic drugs, possibly by enhancing core survival pathways. Interestingly, the authors could not identify the nature of the molecular pathways regulated by PD-L1 signal transduction or the regulatory motifs within its intracytoplasmic domain.^24^

Other indirect experimental evidence has suggested that PD-L1 has signal transduction capacities that contribute to tumor progression by modulating glucose metabolism. Cancer cells actively consume glucose from the tumor environment. In this manner, they strongly inhibit effector T cells that rely on aerobic glycolysis to exert their cytotoxic functions.^88,89^ Interestingly, anti-CTLA-4, anti-PD-1 or anti-PD-L1 antibody treatment restores glucose levels within the tumor environment, suggesting that these immune checkpoint inhibitors regulate glucose metabolism in cancer cells. The authors of these studies demonstrated that in the absence of T cells, PD-L1 directly regulated the metabolism of several cancer cell lines, possibly by signal transduction mechanisms. Therefore, an antibody-mediated PD-L1 blockade in cancer cells inhibited the AKT/mTOR signaling pathway, resulting in the reduced translation of mRNAs encoding glycolytic enzymes.^89^ The same results were achieved by silencing PD-L1, strongly suggesting that PD-L1 itself was the modulator of glycolysis in cancer cells. Nevertheless, no impairment in proliferation or the tumor growth rate was observed in this particular murine sarcoma model.

The regulatory capacities of PD-L1 over the mTOR pathway were demonstrated shortly thereafter in murine B16 melanoma and ID8agg ovarian cancer cell lines ^90^ in the absence of T cells. This further confirmed that PD-L1 possessed signal transduction capacities without needing to engage PD-1. In these murine cancer models, reduced PD-L1 expression altered cancer cell proliferation, especially of B16 cells, which showed decreased proliferation. A transcriptomic study was performed on PD-L1-silenced cancer cells, and several genes differentially regulated by PD-L1 were identified. These genes proved to be mTOR-regulated and involved in autophagy.^90^ These results again confirmed the regulation of the mTOR pathway by PD-L1, although the authors of this study did not address the mechanisms by which PD-L1 exert these effects.

These results clearly suggest that PD-L1 functions via activation of the mTOR-AKT pathway. Thus, the use of mTOR-AKT inhibitors in combination with antibody-based immunotherapies could constitute a rational choice. However, immunotherapies require more than mTOR and AKT inhibitors and anti-PD-L1/anti-PD-1 antibodies. First, we must understand how PD-L1 transduces signals and identify the signalosome associated with PD-L1. The identification of these targets will open the possibilities for targeted combinatorial strategies.

## PD-L1 signaling motifs and anti-interferon functions

Although PD-L1 is currently a major target in medical oncology, surprisingly, very little is known about its intrinsic functions apart from engaging PD-1 on T cells. This can be understood if we consider that the intracellular cytoplasmic part of PD-L1 notoriously lacks any conventional signaling motifs. This has hampered any systematic study on its functions. A thorough search of functional or structural motifs within the PD-L1 intracytoplasmic domain provides very little results. Only MotifFinder produces significant hits, but they are surprisingly related to a domain present in DNA-dependent RNA polymerase beta subunits, as published in our paper by Gato-Canas et al.^25^ (Fig. 2a). This may indicate some convergence towards a particular structural feature. However, the relevance of this observation remains to be determined.

We recently published a study to identify intracellular signaling motifs in PD-L1 and their relevance in cancer cell growth and resistance to interferons.^25^ Hence, to identify potential signal motifs within the intracytoplasmic terminus of PD-L1, we undertook a classical approach by comparing 10 mammalian PD-L1 molecules.^25^ Three well-conserved sequence motifs were identified, termed “RMLDVEKC”, “DTSSK”, and “QFEET” according to the most representative conserved residues^25^ (Fig. 2a). The RMLDVEKC motif in PD-L1 was required for cancer cells to withstand the apoptotic capacities of type I and II interferons. Indeed, this motif was absolutely required to protect against IFNs and provided functionality to the so-called “molecular shield”. Surprisingly, removal of the DTSSK motif significantly enhanced the anti-apoptotic activities of PD-L1, which was also achieved by mutating the lysine residues within these two motifs.^25^ Thus, the DTSSK and lysine residues present in the carboxy terminus are negative regulators of PD-L1 activity. Interestingly, the distal part of the RMLDVEKC motif and the entire DTSKK motif are placed within the RNA polymerase-like motif (Fig. 2a). Our studies confirmed that PD-L1 possesses intrinsic signal capacities that protect cancer cells.^25^ Our observations agree with Azuma et al.^24^ proposing that PD-L1 molecules constitute a molecular shield against pro-apoptotic signals via intracellular signal transduction.

However, how PD-L1 performs its function remains in question. As we demonstrated,^25^ the absence of PD-L1 signal transduction in melanoma cells enhanced STAT3 up-regulation without affecting STAT1 or STAT2 levels after IFNβ stimulation. Indeed, the lack of PD-L1 signal transduction selectively induced STAT3 tyrosine 705 phosphorylation but not that of serine 727, suggesting that PD-L1 also regulates STAT3 phosphorylation.^25^ In the absence of PD-L1, caspases 7 and 9 are strongly up-regulated and required for IFN-dependent apoptosis (Fig. 2b). It is tempting to speculate that PD-L1 signaling motifs recruit adaptor proteins and kinases that may regulate IFN signal transduction and other survival pathways. Proteins belonging to the mTOR signaling pathway may represent good candidates. Ubiquitination in the inhibitory lysine residues may also alter PD-L1 functions in signaling by either affecting its stability and surface expression^26^ or by regulating the recruitment of other signaling components.^25^

Thus, to understand the mechanisms underlying the properties of PD-L1 and identify potential targets, we must first study the intracellular signalosome of PD-L1.

## The PD-L1 signalosome in human cells

**Fig. 3:**
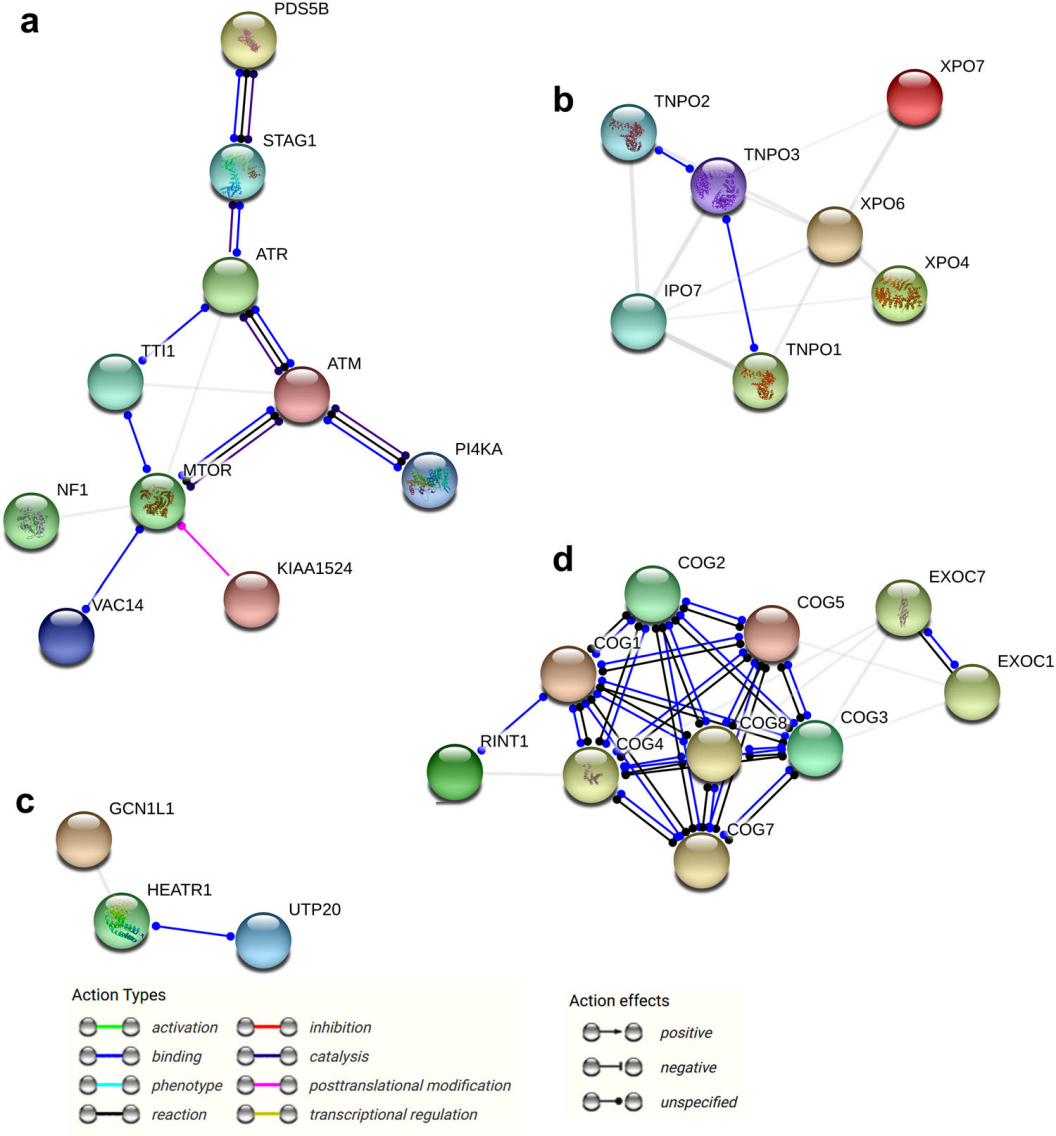
The PD-L1 interactome in human cells. The human PD-L1 interactome was obtained by high-throughput affinity-purification mass spectrometry performed on human 293T cells.^91^ STRING analysis was performed by introducing all the proteins from the interactome (supplementary figure 1) as inputs in STRING (https://string-db.org/) and selecting the Homo sapiens database to detect interactions with a medium confidence of 0.7. The STRING output classified the proteins into four functional groups, a “signalosome” group (**a**), a nuclear import/export group (**b**), a mRNA import/export interactome group (**c**) and a Golgi-ER interactome group (**d**). The most relevant interactomes are (**a**) and (**d**) according to PD-L1 functions and processing in the Golgi-ER. The relationships between proteins are indicated within the graph as action types and action effects

The interactome of PD-L1 in human cells is available from data generated in a recent study by Huttlin et al.^91^ performed using high-throughput affinity-purification mass spectrometry on human 293 T cells. This interactome of PD-L1 was identified using CD274-specific antibodies to capture PD-L1 and associated proteins, followed by identification of these proteins by mass spectrometry (the whole interactome of PD-L1 detected in the study by Huttlin et al. is shown in Supplementary Figure 1 and Supplementary Table 1). The proteins associated with antibody-captured PD-L1 can be classified into four functional protein groups (Fig. 3). The most relevant is the “signalosome” group, comprising several kinases that regulate cell survival and stress/genotoxic responses (Fig. 3a). This group includes mTOR, kinases and regulators of the DNA damage response pathway (ATM, ATR, STAG1, PDS5B, TTL1) and kinases involved in survival and anchor-independence cell growth. This interactome agrees with the current experimental data linking PD-L1 signaling with the regulation of mTOR-AKT and anti-apoptotic responses.^24,88,89^ Moreover, mTOR and these stress-associated kinases most likely form a stable macromolecular complex with PD-L1 in the absence of PD-1 engagement. It is tempting to speculate that some of these kinases are physically associated with the RMLDVEKC or DTSSK motifs either directly or via adaptor proteins.

The second and third groups of proteins are the nuclear import/export group and mRNA import/export group, which are involved in protein and RNA import/export to the cell nucleus (Fig. 3b, c). It is unclear whether these proteins play a relevant role in PD-L1 functions because PD-L1 is a cell membrane protein. These proteins may have been non-specifically co-purified with PD-L1-interacting proteins, as the whole cell interacting proteome published by Huttlin et al. will probably require proper validation on a protein-to-protein basis. The fourth group contains Golgi proteins involved in protein transport and may be associated with PD-L1 during its processing in the ER-Golgi (Fig. 3d).

**Fig. 4:**
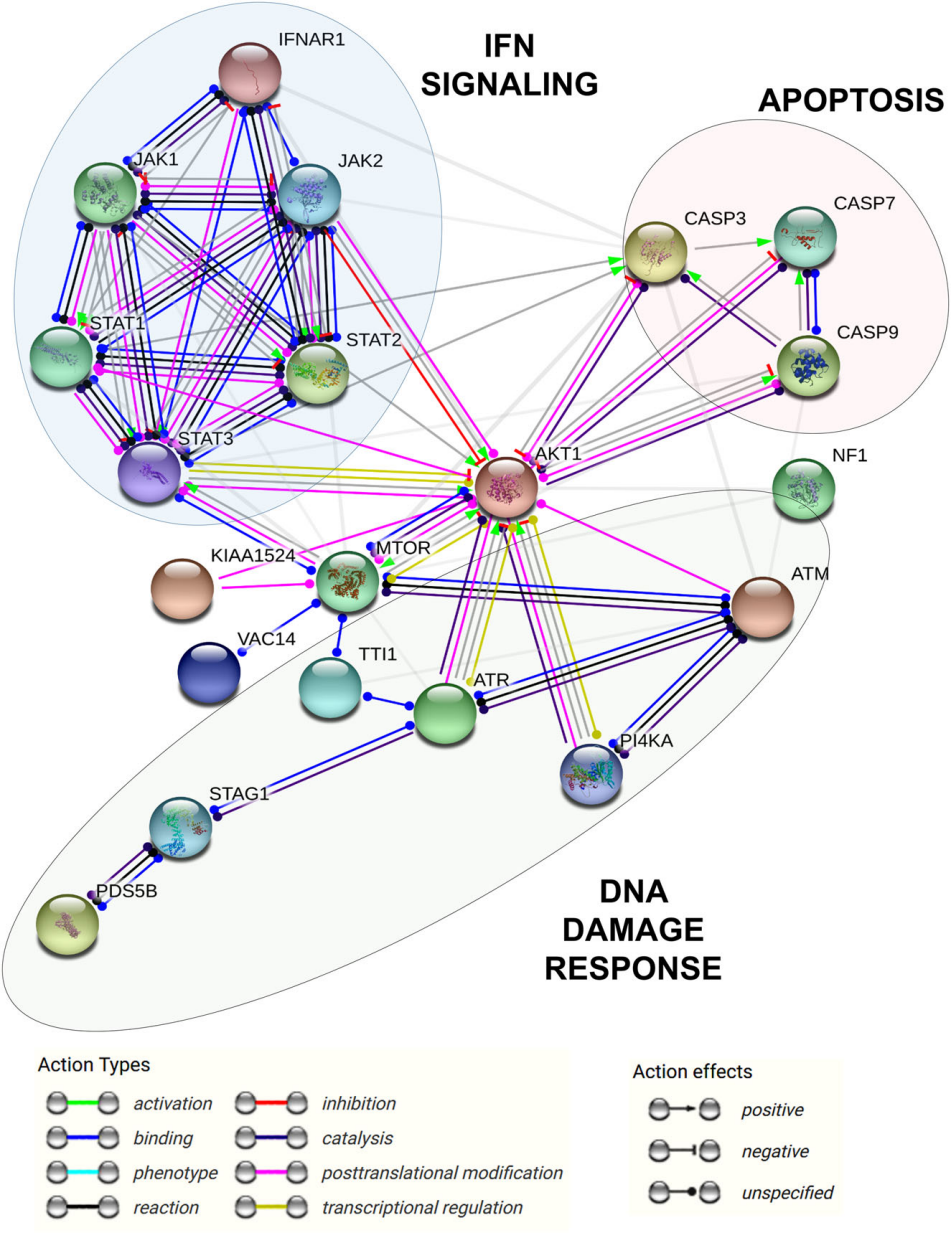
Potential Crosstalk between the interferon signaling pathway and PD-L1 signalosome. STRING analysis of the core mediators of IFN signal transduction. STRING analysis was performed by introducing all the proteins from the interactome (supplementary figure 1 and supplementary Tables 1) together with key regulators of IFN signal transduction (IFNAR1, STAT1, STAT2, STAT3, JAK1, JAK2) and mediators of apoptosis (CASP3, CASP7, CASP9) as inputs (https://string-db.org/) and selecting the Homo sapiens database to detect interactions with a medium confidence of 0.7. According to the functional interactome provided by STRING based on known published molecular interactions, mTOR/AKT plays a central regulatory role coordinating the inhibition of caspases via AKT and its interactions with STATs. Importantly, mTOR/AKT associates and integrates signals from DNA damage response kinases with regulators of Ras signaling, PI4K and c-myc. The relationships between proteins are indicated within the graph as action types and action effects

In our recent publication, we demonstrated that PD-L1 signal transduction regulates interferon responses in cancer cells.^25^ STRING analysis can be used to infer potential molecular crosstalk between the interactome of PD-L1 and mediators of the interferon signaling pathway (Fig. 4). STRING provides potential interactomes among the input proteins by establishing connections and relationships via data mining from published studies. The resulting protein–protein interaction network suggests a molecular crosstalk between PD-L1 and IFN signal transduction pathways at the level of AKT-mTOR. Importantly, these interactions link the regulation of interferon responses and apoptosis by PD-L1 to DNA damage responses via the AKT-mTOR core (Fig. 4).

## PD-L1 signal transduction and anti-cancer immunotherapy. An opportunity for targeted therapies?

PD-L1 undoubtedly inhibits T cell effector activities by binding PD-1.^5,83,84,86,92^ However, it is becoming evident that PD-L1 directly favors cancer cell survival and tumor progression via the modulation of metabolic pathways.^88,90,93^ The therapeutic activities of PD-L1/PD-1-blocking antibodies can be ascribed to two simultaneous mechanisms—reactivation of tumor-infiltrating T cells that would then produce cytotoxic mediators, such as IFNs, and sensitization of cancer cells to IFN-induced apoptosis^25^—directly potentiating cytotoxicity over cancer cells.

Resistance to anti-PD-1 therapy has been described in treated human patients via the selection of cancer cell variants with somatic mutations that inactivate JAK1, or JAK2 or that abrogate β2-microblobulin expression.^81,82^ These cell variants have become intrinsically resistant to interferon-induced apoptosis and do not up-regulate PD-L1. Indeed, disruption of PD-L1/PD-1 binding with antibodies strongly sensitizes cancer cells to apoptosis by IFNs, and only cancer cell variants with mutations that inactivate IFN signal transduction would survive these therapies. In these mutant cells, PD-L1 would not be required to protect against interferons. Extensive evidence shows that cancer cells tend to inactivate IFN signaling via immunoediting.^94,95^ Overexpression of PD-L1 also correlates with tumor progression, possibly by enhancing survival and proliferation pathways in addition to neutralization of the IFN signaling pathway. Interestingly, somatic mutations in human carcinomas occur that disrupt the inhibitory functions of the DTSSK motif. These carcinoma cells express PD-L1 mutants more potent at suppressing IFN signaling,^25^ demonstrating that PD-L1 signaling is crucial for cancer cell survival.

Thus far, PD-L1/PD-1 antibody-mediated blockade therapies have demonstrated good clinical results. A reflection of this is the approval of PD-L1/PD-1 blockade agents for the treatment of an increasing list of cancers. For example, efficacies up to 70% for classical Hodgkin lymphoma,^96^ 60% for advanced Merkel-cell carcinoma,^97^ and good overall results for urothelial carcinoma have been reported.^98^ Indeed, PD-L1/PD-1 immune checkpoint inhibitors are approved for first-line use in melanoma.^99^ However, a significant number of patients do not respond to these therapies. For example, when used as second-line therapies in lung adenocarcinoma patients without selection on the basis of PD-L1 tumor expression, response rates above 20–25% are rarely achieved.^100,102^ Only a subgroup of colorectal cancer patients respond to PD-L1/PD-1 blockades.^103^ This complicates matters for the cancer patient because these therapies are currently very costly, and the demand for these treatments is increasing. Targeted therapies that interfere with the signalosome of PD-L1 with small molecules may provide a more economical alternative than using recombinant antibodies. According to the available data, the mTOR/AKT pathway mediates many PD-L1 functions, and many inhibitors of this signaling axis already exist. For example, rapamycin analogs, such as everolimus,^104^ show synergistic effects with PD-L1/PD-1 blockade therapies.^105^ These combinations are currently being tested in clinical trials to circumvent the resistance to PD-L1/PD-1 blockade therapies.^106^ AKT inhibitors could also be used in combination with PD-L1 blockade, and some are currently being evaluated in clinical trials but not in this combination (MK2206, GSK2141795).

Interestingly, the signalosome of PD-L1 incorporates other potentially targetable kinases that respond to stress and genotoxic responses, such as ATM-ATR kinases. ATM-ATR inhibitors have demonstrated promising results in pre-clinical models with high toxicities toward cancer cells.^107^ Some of these inhibitors are being tested in clinical trials but not in combination with PD-L1/PD-1 blockers.^108,109^ Indeed, no objective reasons for combining them in human clinical trials have existed until now. Here, we propose that using ATM-ATR inhibitors could strongly potentiate PD-L1/PD-1 blockades and reduce the chance of resistance to these therapies. Phosphatidylinositol 4-kinase 2 alpha (PI4KA) inhibitors have been used as cancer therapeutic agents in only a few studies, having demonstrated good results as a radiosensitizing agent.^110^ Co-targeting PI4KA simultaneously with PD-L1/PD-1 blockade could be an attractive approach to reinforce the cytotoxicity of PD-L1 blockade therapies over cancer cells.

KIAA1524 (also known as CIP2A) inhibits protein phosphatase 2A tumor suppressor activity in human neoplastic diseases by favoring the sustained activation of Ras and cellular myelocytomatosis viral oncogene (c-myc).^111^ Its participation in the signalosome of PD-L1 would explain the pro-carcinogenic activities associated with PD-L1 overexpression. Therefore, the combination of erlotinib derivatives with potent inhibitory activities toward CIP2A^112^ with PD-L1/PD-1 blockade therapy represents a rational therapeutic combination to be tested.^113^

## Conclusions

PD-L1 and PD-1 are undoubtedly among the most important therapeutic targets in oncology. Surprisingly, the clinical application of PD-L1/PD-1 blockers has progressed much faster than the study of the basic mechanisms underlying this immunoregulatory interaction. This is especially true for PD-L1, which plays a critical role in cancer cell survival and tumor progression. Recently, we identified two conserved motifs within the intracytoplasmic domain of PD-L1 that mediated the transduction of intracellular signals that inhibit the IFN signaling pathway. Part of the interactome of PD-L1 was also published, uncovering a potential relationship between PD-L1 and the regulation of DNA damage and IFN responses via mTOR as a regulatory node. Hopefully, further understanding on how PD-L1 protects cancer cells from genotoxic damage and immune responses will facilitate more informed choices when designing therapeutic combinations.

## Electronic supplementary material

Supplementary Materials
