# Supplementary material for: The intracellular signalosome of PD-L1 in cancer cells
Source: Signal Transduct Target Ther. 2018 Sep 28;3:26. doi: 10.1038/s41392-018-0022-9 (PMC6160488; doi:10.1038/s41392-018-0022-9)
Supplement: Supplementary file 1 — Supplementary Materials [file 41392_2018_22_MOESM1_ESM.docx]

**Supplementary Materials**

**The intracellular signalosome of PD-L1 in cancer cells**

**David Escors ^1,2^, María Gato-Cañas^1^, Miren Zuazo^1^, Hugo Arasanz^1,3^, María Jesus García-Granda^1^, Ruth Vera^3^, and Grazyna Kochan^1*^**

^1^ Navarrabiomed, Complejo Hospitalario de Navarra, IdISNA, Irunlarrea 3, 31008 Pamplona, Navarra, Spain. ^2^ Rayne Institute, Division of Infection and Immunity, University College London, 5 University Street, WC1E 6JF, London, United Kingdom. ^3^ Oncology Department, Complejo Hospitalario de Navarra, IdISNA, Irunlarrea 3, 31008 Pamplona, Navarra, Spain.

* Corresponding author: Dr Grazyna Kochan. Navarrabiomed, Complejo Hospitalario de Navarra, IdISNA, Irunlarrea 3, 31008 Pamplona, Navarra, Spain. E-mail: grazyna.kochan@navarra.es ; telephone number: +34 848421556;

**Supplementary figure 1**


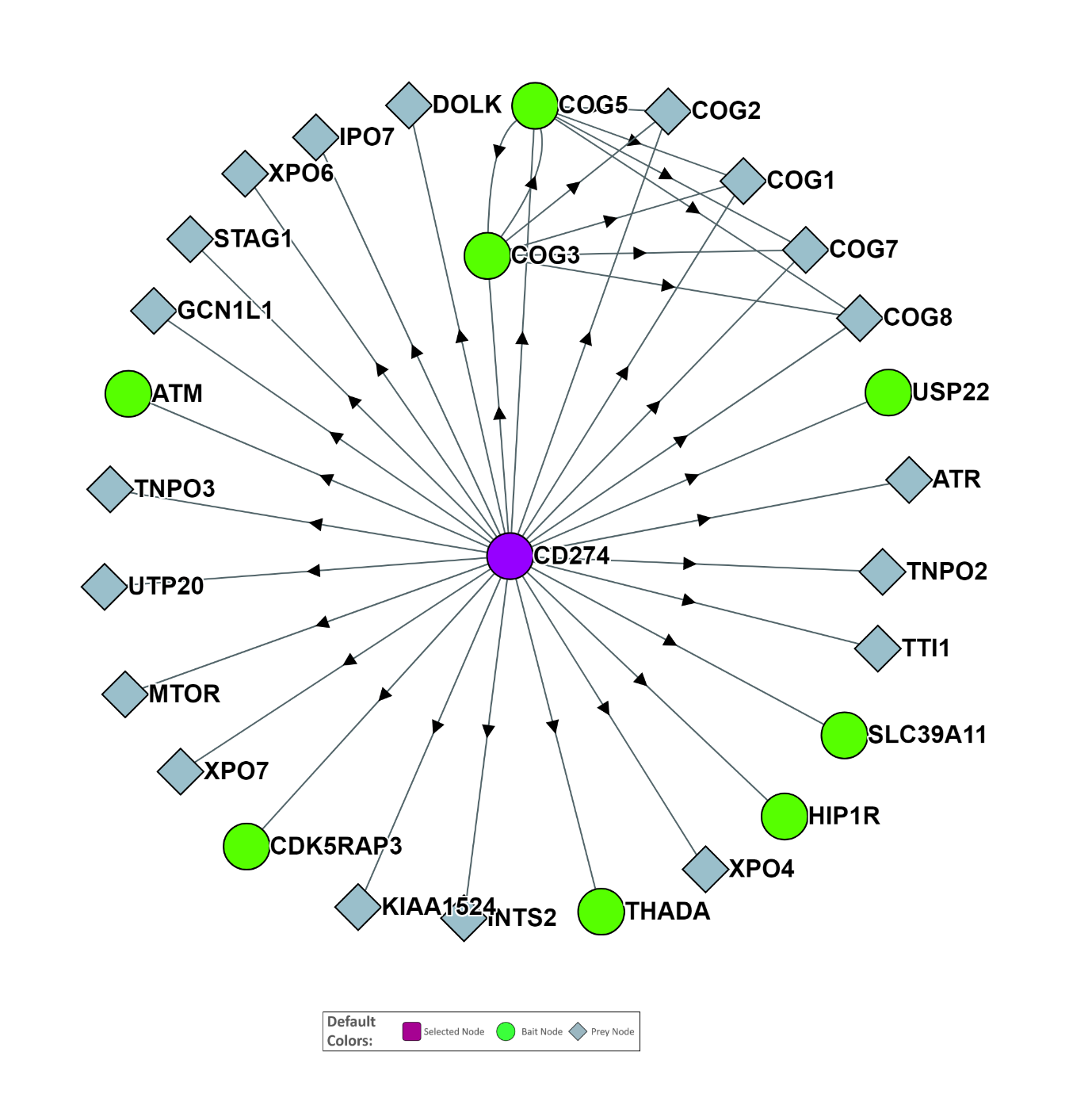


**Supplementary figure 1. Interactome of human PD-L1 in BioPlex by Hutlin et al.** CD274 (PD-L1) is used as an input protein in the BioPlex database (The general link: <http://bioplex.hms.harvard.edu/bioplexDisplay/index.php>) to find the interactome of the PD-L1. From a total of 39 identified proteisn, BioPlex 2.0 provides a CD274 network containing 37 interactions across 28 nodes (excluding self-loops). All intercoms are listed in **Supplementary table S1.**

**Supplementary table S1. Identified intercoms of PD-L1 from BioPlex 2.0 database**

| **Gene name** | **UniprotB** |
| --- | --- |
| SLC39A11 | Q8N1S5 |
| CAND2 | O75155-2 |
| THADA | Q6YHU6-3 |
| PRKDC | P78527 |
| XPO7 | Q9UIA9 |
| UTP20 | O75691 |
| GCN1L1 | Q92616 |
| XPO6 | Q96QU8 |
| CD274 | Q9NZQ7 |
| IPO7 | O95373 |
| ORC4 | O43929 |
| ATR | Q13535 |
| HEATR3 | Q7Z4Q2 |
| TMEM160 | Q9NX00 |
| TNPO2 | Q4LE60 |
| SAAL1 | Q96ER3 |
| TTI1 | O43156 |
| XPO5 | Q9HAV4 |
| COG2 | Q14746 |
| HIP1R | O75146 |
| XPO4 | Q9C0E2 |
| USP22 | Q9UPT9-2 |
| PI4KA | P42356 |
| INTS2 | Q9H0H0 |
| NUP85 | Q9BW27 |
| KIAA1524 | Q8TCG1 |
| CDK5RAP3 | Q96JB5-2 |
| COG1 | Q8WTW3 |
| MTOR | P42345 |
| COG3 | Q96JB2 |
| TNPO3 | Q9Y5L0-3 |
| ATM | Q13315 |
| COG7 | P83436 |
| STAG1 | Q8WVM7 |
| COG8 | Q96MW5 |
| PDS5B | Q9NTI5-2 |
| COG5 | Q9UP83-2 |
| MINPP1 | Q9UNW1-2 |
| DOLK | Q9UPQ8 |

All proteins in Table S1 is obtained from a table output that BioPlex provided in 15^TH^ May 2018
